# The Relationship Between Alcohol and Glycohemoglobin: A Biopsychosocial Perspective

**DOI:** 10.1089/biores.2019.0009

**Published:** 2019-10-03

**Authors:** David A. Wiss

**Affiliations:** Department of Community Health Sciences, Fielding School of Public Health, University of California Los Angeles, Los Angeles, California.

**Keywords:** alcohol, biopsychosocial, diabetes, gender, glycohemoglobin

## Abstract

With the rising prevalence of type 2 diabetes mellitus (T2DM), there is debate regarding biological and psychosocial risk factors. While it is well established that alcohol lowers glycohemoglobin (HbA1c) levels, it is less clear whether alcohol consumption is protective of T2DM. It is also unclear how gender and ethnicity influence the utility of HbA1c screening as a tool for T2DM diagnosis, particularly in the context of alcohol use. This cross-sectional study utilized the National Health and Nutrition Examination Survey 2013–2014 dataset and was restricted to adults 20 years and older, nonpregnant, and not on antihypertensive medication (*n* = 4299) to evaluate the relationship between alcohol use and HbA1c. A multilinear regression model controlled for gender, ethnicity, education level, body mass index, and age. After controlling for covariates, both moderate (*β* = −0.073; *p* = 0.033) and heavy drinking (*β* = −0.167; *p* < 0.001) are associated with reduced HbA1c levels. Additionally, female gender is a significant negative predictor of HbA1c (*β* = −0.052; *p* = 0.024) and all ethnic groups have higher levels of HbA1c compared with non-Hispanic whites. Plausible biological mechanisms are discussed. The clinical utility of HbA1c as a screening tool for T2DM without considering alcohol use, gender, and ethnicity may lead to diagnostic errors. Individualized approaches and focused efforts toward health equity are needed to address rising rates of T2DM.

## Introduction

### Diabetes

The prevalence of type 2 diabetes mellitus (T2DM) has steadily increased in recent years.^1^ The National Health and Nutrition Examination Survey (NHANES) from 2011 to 2012 document a range between 12% and 14% among U.S. adults, with higher prevalence in non-Hispanic black, non-Hispanic Asian, and Hispanics.^2^ Several studies suggest that socially disadvantaged groups have higher rates of T2DM^3^ as well as poorer outcomes.^4^ A recent review article identified three categories of risk factors for T2DM: (1) socioeconomic status (SES), (2) psychosocial stress, and (3) sleep deprivation and work stress, all of which are more pronounced in females.^5^ Estimates suggest that the prevalence of undiagnosed T2DM is ∼30% of those diagnosed.^6^ Despite better awareness and more effective medications, the overall percentage of people achieving glycemic control has not improved in the last three decades.^7,8^ It is of great public health significance to reverse this trend.

Glycohemoglobin (HbA1c) was discovered in the 1960s and has been used as a useful screening tool for diabetes and prediabetes since 2010.^9^ HbA1c is a naturally occurring, nonenzymatic product from exposure of hemoglobin to glucose, reflecting the average plasma glucose concentration over the life span of the red blood cell (∼100–120 days). NHANES data show higher HbA1c in males than females,^10^ which is consistent with men's higher prevalence of T2DM.^6,11^ A recent meta-analysis concluded that HbA1c values are higher in blacks, Asians, and Latinos compared with whites.^12^ Interestingly, biomarkers of iron deficiency have been identified as confounders because they may increase HbA1c levels.^13,14^ Recently, authors have proposed a reinterpretation of HbA1c levels that incorporate ethnicity, gender, age, and body mass index (BMI) differences.^15^ It is also likely that alcohol use should be considered in the clinical interpretation of HbA1c. The current investigation hypothesizes that alcohol consumption lowers HbA1c, which has the potential to impact T2DM diagnosis.

### Alcohol

Alcohol consumption continues to rise in the United States.^16,17^ Excessive drinking has been linked to the metabolic syndrome, a cluster of conditions, including obesity, hypertension, and T2DM.^18^ Several recent studies have shown that alcohol consumption is inversely associated with HbA1c,^19–21^ including studies using NHANES protocols in the United States^22^ and Korea.^23^ Research has shown that alcohol decreases concentrations of HbA1c levels independent of plasma glucose.^19^ The biochemical relationship between alcohol consumption and HbA1c^23^ is not well understood. Many researchers have concluded that alcohol consumption is protective against T2DM,^24^ however, this is not true for binge drinking.^25^ Evidence from NHANES suggests that alcohol consumption increases insulin sensitivity, which may explain its ability to lower HbA1c.^26^ Given the inconsistencies in clinical practice around the recommendations for alcohol use in blood sugar control, this investigation aims to clarify the relationship between alcohol and HbA1c by controlling for known confounders. Alcohol has been associated with poorer T2DM self-care,^27^ yet it remains unclear if alcohol is metabolically helpful for those with T2DM.

### Alcohol and gender

Alcohol consumption is higher in males than females.^28^ Men's increased likelihood of drinking has been described as one of the few universal gender differences in human behavior, with men being more than twice as likely to report heavy episodic drinking.^29^ A recent report suggests that the gender gap is closing and described an “emerging female alcohol epidemic.”^30^ Gender refers to the adaptation of social, cultural, and behavioral attributes “prescribed” by society, and the associated environmental and sociocultural defined roles for men and women. Several reports have implicated the traditional male gender role with drunkenness,^31^ whereas intoxicated women are seen as breaking traditional codes of femininity.^32^ There is a need for global alcohol research to incorporate multidimensional and intersecting relational theories of gender,^33^ which may differ across countries and cultures.^34^

### Alcohol and ethnicity

Understanding variations in alcohol use among ethnic groups relies on a complex interplay of psychological, historical, cultural, and social factors, which can also impact help seeking and subsequent clinical diagnosis. Some authors believe there are inaccurate stereotypes about Hispanics, Indians, and Asians, which perpetuate perceived differences.^35^ In general, higher rates of drinking are reported in Native Americans and Hispanics, whereas whites and Native Americans have a greater risk of developing an alcohol use disorder (AUD), and blacks and Hispanics have higher rates of persistent dependence.^36^ In one study, difficulties at home have been associated with an increased risk of heavy drinking for non-Hispanic blacks and Hispanics.^37^ AUD has been associated with poorer health in black women compared with white women.^38^

Differences in drinking patterns across ethnicities can be related to immigration experiences, discrimination, economic and neighborhood disadvantage, as well as variations in alcohol-metabolizing genes. Social disadvantage has been linked to alcohol-attributable harms such as violence/injury and various disease states,^39,40^ as well as social consequences.^41^ Interestingly, a protective effect of alcohol on the metabolic syndrome has been shown for whites,^26^ whereas NHANES data found that among current drinkers, Mexican and non-Hispanic blacks have increased risk of liver damage.^42^ Overall, the literature suggests that differential patterns in alcohol-attributable injury, particularly for blacks and Hispanics cannot be easily explained by ethnic differences in consumption patterns^43^ pointing toward social determinants of health (SDOH) framework considering other upstream influences on health.

### Alcohol and SES

The link between SES and alcohol consumption is inconsistent. Increased income has been positively associated with alcohol consumption and some forms of abuse.^44^ Higher levels of education have been associated with increased drinking.^45^ Some authors have suggested a need to consider different dimensions of drinking such as bingeing versus casual drinking^46^ to capture the effects of SES on alcohol-related outcomes.^47^ While higher SES predicts more drinking, lower SES groups are more likely to drink at extreme levels (highest threshold of heavy episodic drinking).^47^ Systematic reviews have concluded that while SES does not predict consumption of alcohol, low SES is associated with higher mortality risk.^48^ A large dataset from Scotland showed that SES is a significant modifier in the relationship between alcohol consumption and associated harm.^39^

Education is a well-established proxy measure for SES. Higher levels of education are consistently linked to lower levels of overall biological risk.^49^ NHANES data have shown that lower levels of education are associated with higher levels of HbA1c.^10^ Higher levels of education have been linked to lower rates of obesity.^50^ Current data suggest a positive association with education levels and alcohol consumption.^45,51^

### Body mass index

NHANES data also show a strong association between percent body fat and HbA1c.^52^ The prevalence of T2DM increases with rising weight classes.^53^ Adipose tissue releases a wide range of bioactive mediators (e.g., adipokines such as leptin) that have been shown to contribute to insulin resistance, the core feature of T2DM.^54^ The fact that women have more adipose tissue than men has been cited as a potential explanation for sex differences in insulin function.^5^ Pooled NHANES data between 1988 and 2014 indicate that rising levels of adiposity may explain the increased incidence of T2DM.^55^ The relationships between alcohol consumption and obesity are mixed. NHANES data link frequent or heavy alcohol consumption with greater odds of being obese,^56^ while moderate drinking appears protective.^57^

### Age

Several studies have documented graded increases in HbA1c with older age.^58,59^ In a Korean sample, increasing age has been associated with a discordance between diagnostic criteria for T2DM based on fasting glucose and HbA1c.^60^ Investigators using NHANES data in the United States have shown that HbA1c cutoffs for diabetes (6.5%) and prediabetes (5.7%) can be misleading if overlooking age effects.^61^

## Theoretical Framework

The biopsychosocial perspective was originally proposed by Dr. George Engel in 1977 based on his dissatisfaction with the reductionistic biomedical model of illness.^62^ A central theme with this approach is the use of divergent conceptual models (biological, psychological, social) emphasizing multicausality in understanding disease. Other authors have agreed on a need for an integrated vision of health and disease that does not focus on a single root cause, but instead looks at an entire system.^63^ Given that social conditions are known upstream drivers of disease putting individuals “at risk of risk,”^64^ the current investigation will conceptualize the role of education (proxy for socioeconomic disadvantage associated with minority groups) as an important psychosocial predictor of HbA1c in the model. In line with the biopsychosocial framework, both biological and social determinants of HbA1c are investigated by this study.

## Methods

### Sample

NHANES 2013–2014 (*n* = 5769) combines interviews (demographic, socioeconomic, dietary, etc.) with physical examinations (including laboratory tests). NHANES samples 5000 people across 15 different U.S. counties during each 2-year period, selected to represent the U.S. population of all ages (dataset).^65^ NHANES oversamples people over 60, African Americans, Asians, and Hispanics to produce reliable (weighted) statistics. Individuals using antihyperglycemic medications (*n* = 642) were excluded from the study due to altered HbA1c status.^22^ Pregnant women (*n* = 65) were also excluded due to HbA1c levels being significantly lower in early and late pregnancy.^66^ Health interviews are conducted in respondent's homes and measurements are conducted in equipped mobile centers, which travel throughout the country (dataset).^65^ Because this dataset is publicly available, free of personal information, and consent has already been given through NHANES, the study is exempt from Institutional Review Board approval.

### Measures

The outcome variable HbA1c is expressed as a percentage of HbA1c in whole blood specimens (mmol/mol). The Tosoh Automated Glycohemoglobin Analyzer HLC-723G8 uses nonporous ion exchange, high-performance liquid chromatography and microcomputer technology to quickly and accurately measure HbA1c. This value offers an accurate indication of the participants' diabetic control over the past 2 to 3 months. In the final analytic sample, the mean HbA1c equals 5.52%.

The key predictor is alcohol use, operationalized by the following categories: never (reference), former, light (<3 drinks/week), moderate (up to 2 drinks/day for men; 1 drink/day for women), heavy (more than moderate). Gender is coded as male (reference) or female. Ethnicity includes the following: non-Hispanic white (reference), Mexican American, other Hispanic, non-Hispanic black, non-Hispanic Asian, and other/multi (which is chosen by participant). Education includes the following: less than 9th grade (reference), 9th—11th grade, high school/general education diploma, some college/associate's degree, and college grad or more. BMI is operationalized as a categorical variable following the Western standards: underweight (<18.5; reference), normal (18.5–24.9), overweight (25–29.9), and obese (>30). Age (years) has been categorized into: 20–34 (reference), 35–49, 50–64, and 65+.

### Data analysis

All data analysis was performed using STATA version 15.0.^67^ HbA1c has 5392 observations, 377 are missing (6.5%). The distribution of HbA1c is skewed slightly to the right, but previous NHANES reports have shown that using the logarithm leads to identical conclusions^22^ so the outcome was left untransformed. For alcohol use, 678 are missing (11.8%) due to the fact that alcohol data were collected through the mobile exam center (MEC), which has higher rates of missing data (not all participants visited the MEC). BMI has 249 missing values, and seven are missing from education. All missing data were dropped. See Supplementary Figure S1 for derivation of final analytic sample (*n* = 4299) and sensitivity analysis of missing data. A multilinear regression model was used, including all of the predictors.

## Results

Bivariate analyses reveal a dose-dependent decreasing effect of alcohol use on HbA1c, summarized in Table 1. Next, weights were applied before running the multilinear regression with all covariates. Results from the final linear regression model are summarized in Table 2. After adjusting for confounders, increasing alcohol consumption predicts decreasing HbA1c levels for moderate (*β* = −0.073; *p* = 0.033) and heavy (*β* = −0.167; *p* < 0.001) drinkers, relative to never drinkers. Female gender is a significant negative predictor of HbA1c (*β* = −0.052; *p* = 0.024) and all ethnic groups have higher levels of HbA1c compared with non-Hispanic whites in the full model. Relative to less than 9th grade education level, college graduates have lower levels of HbA1c (*β* = −0.176; *p* = 0.005), controlling for covariates. Relative to underweight, overweight (*β* = 0.120; *p* = 0.040) and obese (*β* = 0.285; *p* < 0.001) are associated with a positive effect on HbA1c, controlling for covariates. Finally, increasing age is significantly associated with increasing HbA1c in a dose-dependent fashion.

**Table 1.** Unweighted Bivariate Relationship Between Alcohol Use and Glycohemoglobin

	HbA1c summary statistics		
Alcohol use	Mean	SD	Frequency (%)
Never	5.60	0.70	619 (14.4)
Former	5.71	0.87	660 (15.4)
Light	5.50	0.64	2002 (46.6)
Moderate	5.42	0.52	707 (16.4)
Heavy	5.31	0.43	311 (7.2)

NHANES 2013–2014 ages 20+ nonpregnant and not on antihyperglycemic medication (*n* = 4299).

HbA1c, glycohemoglobin; NHANES, National Health and Nutrition Examination Survey; SD, standard deviation.

**Table 2.** Multilinear Regression with Outcome Glycohemoglobin

Predictor	*β*	95% CI	*p*
Alcohol use			
Never			
Former	0.065	(−0.065 to 0.194)	0.303
Light	−0.001	(−0.069 to 0.067)	0.972
Moderate	−0.073	(−0.139 to −0.007)	0.033
Heavy	−0.167	(−0.227 to −0.106)	<0.001
Gender			
Male			
Female	−0.052	(−0.095 to −0.008)	0.024
Race/ethnicity			
Non-Hispanic white			
Mexican American	0.133	(0.068 to 0.197)	0.001
Other Hispanic	0.183	(0.094 to 0.273)	0.001
Non-Hispanic black	0.193	(0.123 to 0.264)	<0.001
Non-Hispanic Asian	0.254	(0.199 to 0.310)	<0.001
Other/multi	0.120	(−0.020 to 0.259)	0.087
Education			
Less than 9th grade			
9th–11th grade	−0.050	(−0.172 to 0.072)	0.393
High school/GED	−0.088	(−0.214 to 0.038)	0.158
Some college/AD	−0.113	(−0.246 to 0.020)	0.090
College grad+	−0.176	(−0.291 to −0.061)	0.005
BMI			
Underweight			
Normal	0.037	(−0.074 to 0.147)	0.489
Overweight	0.120	(0.006 to 0.233)	0.040
Obese	0.285	(0.186 to 0.384)	<0.001
Age (years)			
20–34			
35–49	0.188	(0.135 to 0.241)	<0.001
50–64	0.364	(0.328 to 0.401)	<0.001
65+	0.533	(0.482 to 0.585)	<0.001
Constant	5.154	(4.994 to 5.314)	<0.001

NHANES 2013–2014 ages 20+ nonpregnant and not on antihyperglycemic medication (*n* = 4299).

AD, associate's degree; BMI, body mass index; CI, confidence interval; GED, general education diploma.

## Discussion

Higher levels of drinking predict lower levels of HbA1c in a dose-dependent fashion, after controlling for gender/sex, ethnicity/race, education, BMI, and age. Assuming the final model provides an adequate prediction of HbA1c, a 40-year-old normal-weight white female with a college degree and heavy drinking patterns could be expected to have HbA1c equal to 4.98% (5.154_constant_ – 0.167_heavy drinker_ – 0.052_female_ + 0_white_ – 0.176_college grad_ + 0.037_normal weight_ + 0.188_35–49_), which is very close to the accepted national mean of 5.0%.^65^ Meanwhile, a 55-year-old overweight black male who is a high school graduate and light drinker could be expected to have HbA1c equal to 5.74% (5.154_constant_ – 0.001_light drinker_ + 0_male_ + 0.193_black_ – 0.088_high school_ + 0.120_overweight_ + 0.364_50–64_), which is considered prediabetic. Whether these differences are due to underlying SDOH or biological influences could be debated. A point/counterpoint on the importance of racial differences in the interpretation of HbA1c has been published.^68^

A biopsychosocial framework considers all possible streams of causation (biological, psychological, social); however, it is implausible to fully specify a regression model based on data limitations as well as multicollinearity. The current model is only able to capture a portion of what an integrated biopsychosocial perspective aspires to do (*R*^2^ = 0.184). For example, HbA1c is highly influenced by dietary patterns, which was not included in the current model. The social determinants of nutrition behavior and obesity have been well described.^69^ Meanwhile, a recent systematic review recognizes several of the limitations and research gaps in efforts to describe the link between SES and BMI through psychosocial factors.^70^ NHANES data show that alcohol consumption alters daily dietary pattern by displacing carbohydrate, protein, and fat.^71^ It has been suggested that hypoglycemia resulting from alcohol in diabetic patients may be in response to carbohydrate intake patterns.^72^ It is also common for heavy drinkers to have poor nutrition and in some cases become severely malnourished.^73^ Given that social class predicts dietary quality,^74^ future models should include nutrition, and control for other possible measures of SES. One plausible theory could be that since alcohol consumption is positively associated with income and education, the negative effects of alcohol could be capturing SES, rather than the direct effects of alcohol on hormones and organ systems.

While all covariates have predictive power in the model, the impact of alcohol on HbA1c has not received adequate attention amidst the T2DM epidemic. Several NHANES studies examining HbA1c as the outcome have been conducted, but alcohol use is seldom a predictor.^7,8,10,52,55^ Similarly, many major T2DM studies fail to control for alcohol use.^1^ This suggests that the impact of alcohol on HbA1c levels is not well known in some disciplines. One study found consistent differences in individual levels of HbA1c relative to other glucose markers, which investigators were not able to explain.^75^ The complex relationship between alcohol and HbA1c will need to be evaluated in more complex models,^76,77^ including a wider range of both biological and social predictors. This may aid in public health efforts to achieve better glycemic control in the population.

### Plausible biological mechanisms

A biological model should emphasize the direct effect of alcohol on blood glucose, which can vary across different genetic variants and sexes.^78^ Factors, such as red blood cell survival, cellular glucose balance, and nonglycemic determinants of hemoglobin glycation (i.e., genetics), also vary by race.^79^ *In vitro* ethanol exposure significantly decreases hemoglobin content and concentration in red blood cell cytoplasm^80^ suggesting that the direct impact of alcohol on hemoglobin might partially explain the overall effect of alcohol on HbA1c. Shortened red cell survival has been shown to falsely lower HbA1c values in the face of hyperglycemia.^81^ Meanwhile, it has been suggested by the World Health Organization (WHO) that alcoholism is associated with increased HbA1c through increase in glycation.^82^ Therefore, discerning between the acute effect of alcohol versus chronic alcoholism over the life course might elucidate effects of alcohol on organ systems (i.e., liver), which in turn impact red cell survival, glucose homeostasis, and subsequent HbA1c levels. More research is needed on differing levels of glycation for a given glucose level across ethnic groups as well as drinking status over time.

On days after alcohol consumption, hypoglycemia has been observed in T1DM.^83^ Next-day hypoglycemia is a plausible mechanism by which HbA1c is lowered by alcohol. Individuals self-administering insulin should be aware of potential hypoglycemia on days after drinking.^83^ A T2DM case study has shown that the “day after” effect of reduced blood glucose was only observed after drinking red wine.^84^ Polyphenols (e.g., resveratrol) found in wine have been shown to have antihyperglycemic effects^85^ and it has been suggested that the protective effects of wine in relation to T2DM are more pronounced than beer or spirits.^86^ Future research should investigate the possibility that polyphenolic compounds in alcoholic beverages play a role in reduced HbA1c, and how the type of drink may confound this relationship.

Differences in body water (women have less) have been cited as a potential explanation why women are more sensitive to the effects of alcohol.^87^ Importantly, women have ∼12% lower hemoglobin levels in venous blood, which is also true of many species of mammals, birds, and reptiles indicating that it is a sex-related phenomenon (related to hormones).^88^ In a large Korean sample, women have higher values of HbA1c compared with men, attributable to lower hemoglobin levels in women but after adjustment for hemoglobin level, HbA1c remained consistently lower in women compared with men,^89^ consistent with data presented in this study. Many studies have identified sex differences in the relationship between alcohol and glucose status^90^ but cannot delineate if effects are due to sex or gender. A biopsychosocial model attempts to consider both streams of influence.

Strong correlations between alcohol and HbA1c have been weakened by the use of insulin and oral medications^22^ suggesting a distinct biological pathway involving insulin. Additionally, alcohol inhibits gluconeogenesis in the liver and decreases glycogenolysis, lowering blood sugar.^91^ A review article concluded that acute ethanol ingestion causes insulin resistance while chronic ethanol intake improves insulin sensitivity.^92^ Another review stated that alcohol increases insulin secretion, therefore, appears to improve glucose tolerance.^93^ While acute alcohol consumption appears to improve insulin action without affecting insulin secretion in T2DM patients,^94^ less is known about the long-term effects of alcohol on insulin activity. Interestingly, sober individuals with AUD have shown blunted responses to blood glucose infusions, suggesting that previous alcohol abuse may have lasting effects on glucose homeostasis.^95^ Biopsychosocial models pose many challenges given the need to incorporate biological predictors (e.g., iron status) that are not easily obtained, and to include social theories that look beyond mechanistic explanations.

### Summary

Universal cut points for HbA1c may not be applicable across all ethnic groups to properly identify individuals at risk.^96^ Reliance on HbA1c as the sole criteria for T2DM diagnosis creates potential for systematic classification error.^79^ Flawed clinical decisions leading to misdiagnosis and over- or undertreatment of diabetes and prediabetes has major public health significance^97^ and may even contribute to health disparities across ethnic groups. Based on the evidence presented herein, it appears that considering alcohol consumption patterns into the interpretation of clinical HbA1c tests may be of value, as abnormal amounts of alcohol may reduce the diagnostic efficiency of the test. Glucose tolerance tests may be more appropriate when alcohol consumption is high. Furthermore, given the potential for other medical complications, such as disturbances in fat metabolism, nerve damage, and eye disease,^98^ the recommendation of alcohol consumption to T2DM patients to improve HbA1c does not appear justified. Decreasing alcohol consumption has actually been shown to improve weight loss among individuals with T2DM.^99^ A 2018 WHO report highlights that alcohol-attributable disease burden continues to be extraordinarily large^100^ and does not appear to be remitting.

### Limitations

The biopsychosocial perspective aims to understand multicausality, yet the current cross-sectional study cannot infer any direct causation. Operationalization of the alcohol variable was averaged per day, which does not capture the potential effects of infrequent heavy episodic drinking. Therefore, examining differences in drinking patterns (e.g., binge drinking) or type of beverage was not possible. The current study did not control for nutrition, iron status, or exercise, all of which have been shown to have direct effects on HbA1c. The study did not control for smoking status, which has been shown to raise HbA1c.^101^ The analysis did not control for insulin or fasting plasma glucose, both of which have a direct influence on HbA1c. Furthermore, dropped data were not missing at random (Supplementary Fig. S1). Finally, generalizability outside of the United States may be limited.

### Future Directions

Future investigations should take into account the dose of ethanol, previous drinking pattern and frequency, and durations of ethanol consumption, as well as abstention. The type of alcoholic beverage (e.g., wine) may prove to be determinant, and the social correlates of drink type should also be considered. More research is needed to describe HbA1c status in former drinkers to separate those who quit for personal preference versus those who stop drinking due to AUD or organ complications associated with excess use. Future studies should examine potential mediation by insulin in the relationship between alcohol and HbA1c. Current data appear to support an initial hyperinsulinemic effect followed by a hypoinsulinemic and hypoglycemic effect.^102^ There are data to suggest that alcohol lowers fasting insulin^103^ and other reports of the opposite, therefore this needs to be investigated and clarified, both in cross-sectional studies and controlled trials. It will be critical to discern between the effects of acute versus chronic alcohol on glycemic outcomes, which at this time is not clear.

## Conclusions

Overall, the published literature has documented that serum glucose levels have been rising in the United States, leading to an increased incidence of T2DM. Future diabetes screening using HbA1c as criteria should consider alcohol consumption, as well as gender/sex and possibly race/ethnicity. Evidence presented herein and elsewhere suggest that nonglycemic factors, including demographic predictors should be taken into consideration when screening for T2DM. After controlling for gender, ethnicity, education, BMI, and age, alcohol use is a significant negative predictor of HbA1c levels. This may lead to misleading clinical conclusions based on a falsely lowered HbA1c, which may lead to undertreatment. Personalized medicine and sustained efforts toward health equity are needed to adequately address rising rates of T2DM and may require a biopsychosocial perspective.

## Supplementary Material

Supplemental data

## Abbreviations Used

AUD: alcohol use disorder
BMI: body mass index
CI: confidence interval
HbA1c: glycohemoglobin
MEC: mobile exam center
NHANES: National Health and Nutrition Examination Survey
SD: standard deviation
SDOH: social determinants of health
SES: socioeconomic status
T2DM: type 2 diabetes mellitus
WHO: World Health Organization
