# Supplementary material for: The Relationship Between Alcohol and Glycohemoglobin: A Biopsychosocial Perspective
Source: Biores Open Access. 2019 Oct 3;8(1):146–54. doi: 10.1089/biores.2019.0009 (PMC6776959; doi:10.1089/biores.2019.0009)
Supplement: Supplemental data [file Supp_FigS1.pdf]

Supplementary Data

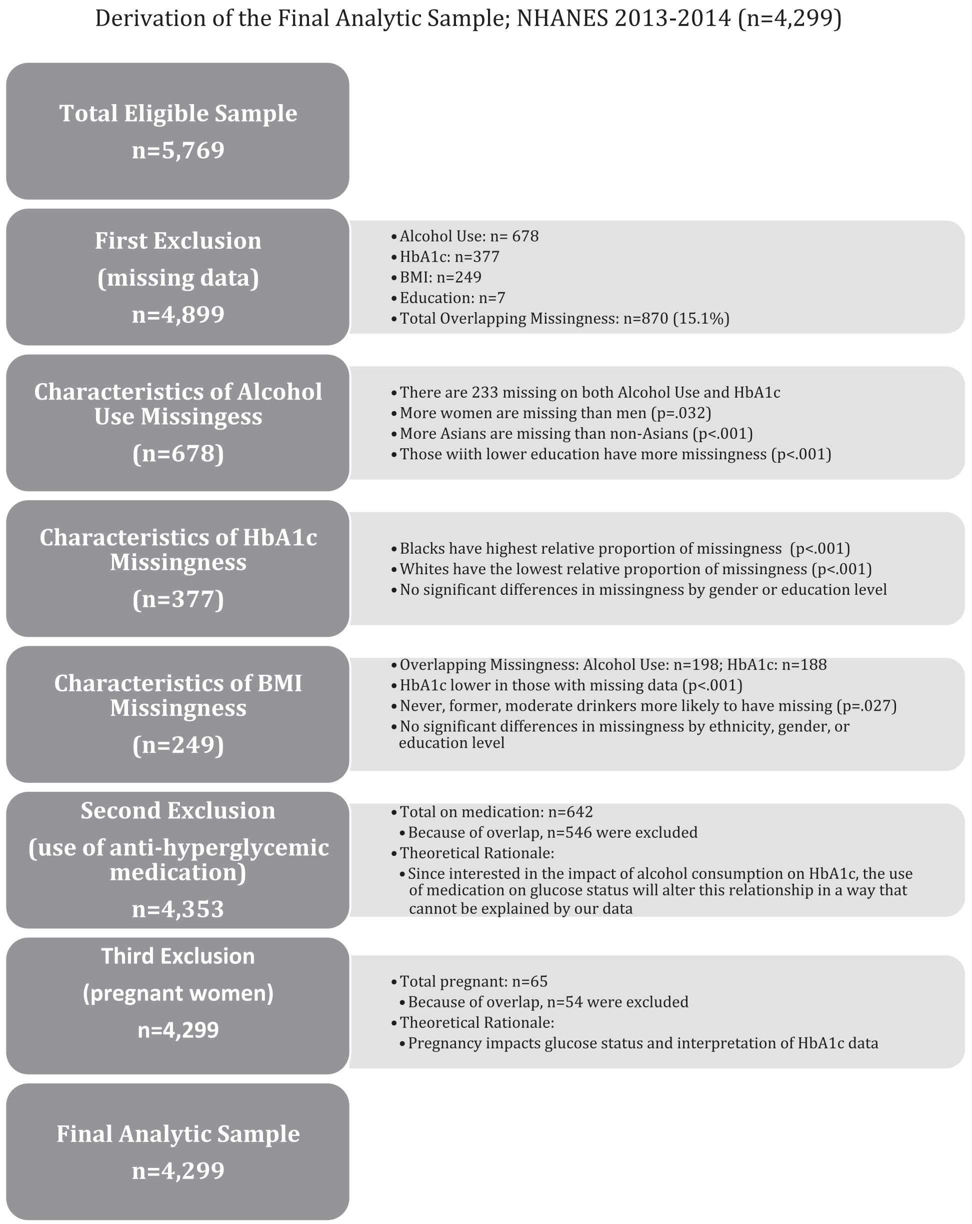

SUPPLEMENTARY FIG. S1. Derivation of the final analytic sample; National Health and Nutrition Examination Survey 2013–2014 ($n = 4299$). BMI, body mass index; HbA1c, glycohemoglobin.
